# A noninvasive swallowing measurement system using a combination of respiratory flow, swallowing sound, and laryngeal motion

**DOI:** 10.1007/s11517-016-1561-2

**Published:** 2016-09-24

**Authors:** Naomi Yagi, Shinsuke Nagami, Meng-kuan Lin, Toru Yabe, Masataka Itoda, Takahisa Imai, Yoshitaka Oku

**Affiliations:** 1 Department of Neurology, Graduate School of Medicine, Kyoto University, 54 Shogoin-Kawaharacho, Sakyo-ku, Kyoto, 606-8507 Japan; 2 Clinical Research Center for Medical Equipment Development (CRCMeD), Kyoto University Hospital, 54 Shogoin-Kawaharacho, Sakyo-ku, Kyoto, 606-8507 Japan; 3 Department of Physiology, Division of Physiome, Hyogo College of Medicine, 1-1 Mukogawa-cho, Hyogo Nishinomiya, 663-8501 Japan; 4 Murata Manufacturing Co., Ltd., 1-10-1, Higashikotari, Nagaokakyo, Kyoto, 617-8555 Japan; 5 Wakakusa Tatsuma Rehabilitation Hospital, 1580 Oaza-tatsuma, Daito, Osaka, 574-0012 Japan; 6 Ashiya Municipal Hospital, 39-1 Asahigaoka-cho, Ashiya, Hyogo, 659-0012 Japan

**Keywords:** Swallowing, Dysphagia, Deglutition apnea, Coordination between swallowing and breathing

## Abstract

The assessment of swallowing function is important for the prevention of aspiration pneumonia. We developed a new swallowing monitoring system that uses respiratory flow, swallowing sound, and laryngeal motion. We applied this device to 11 healthy volunteers and 10 patients with dysphagia. Videofluoroscopy (VF) was conducted simultaneously with swallowing monitoring using our device. We measured laryngeal rising time (LRT), the time required for the larynx to elevate to the highest position, and laryngeal activation duration (LAD), the duration between the onset of rapid laryngeal elevation and the time when the larynx returned to the lowest position. In addition, we evaluated the coordination between swallowing and breathing. We found that LAD was correlated with a VF-derived parameter, pharyngeal response duration (PRD) in healthy subjects (LAD: 959 ± 259 ms vs. PRD: 1062 ± 149 ms, *r* = 0.60); however, this correlation was not found in the dysphagia patients. LRT was significantly prolonged in patients (healthy subjects: 320 ± 175 ms vs. patients: 465 ± 295 ms, *P* < 0.001, *t* test). Furthermore, frequency of swallowing immediately after inspiration was significantly increased in patients. Therefore, the new device may facilitate the assessment of some aspects of swallowing dysfunction.

## Introduction

Dysphagia, or swallowing difficulty, is the medical term for a condition in which the swallowing process is disrupted and eating ability is impaired. Patients with dysphagia can be at higher risk of pulmonary aspiration and subsequent aspiration pneumonia. According to WHO report in 2012, pneumonia was at third rank among causes of death in the world (World Health Organization—Fact sheets of Media Centre, The top 10 causes of death, http://www.who.int/mediacentre/factsheets/fs310/en/), and the majority of pneumonia cases in the elderly population are associated with aspiration. Swallowing abnormality may also contribute to exacerbations of pulmonary diseases [4, 10, 13, 24, 33, 43, 44]. Recurrence of aspiration pneumonia frequently occurs if the underlying swallowing problems have not been properly treated. Therefore, the assessment of swallowing function and early intervention are critical for preventing the occurrence and recurrence of aspiration pneumonia.

 There are several assessment methods that can be applied to evaluate patients with dysphagia. The two widely used bedside swallow assessment tests, repetitive saliva swallowing test (RSST) and modified water swallowing test (MWST), lack quantitative analyses. Currently, videofluoroscopy (VF) and videoendoscopy (VE) are considered the gold standard for evaluating dysphagia. However, VF cannot be conducted frequently since X-ray exposure may endanger the health of patients as well as medical staff. In addition, VF cannot be done outside the medical facility. VE is more portable than VF; however, specifically trained medical doctors (or dentists) must be available to diagnose the findings on site. Swallowing sound and motion analyses are alternative swallowing assessment techniques [1, 5, 22, 34]. However, in that motion analysis, ‘motion’ does not refer to that of the vocal cord, but rather it refers to the elevation of the larynx that causes the downward motion of the epiglottis to cover the airway to protect it during swallowing. Although they are safe, relatively simple, and easily repeatable, these techniques need to process acoustic or kinetic signals obtained by specially designed sensor devices, typically a laryngeal microphone or an accelerometer. Therefore, many researchers have developed algorithms to process these signals for the assessment of swallowing function [8, 22, 27, 36, 42]. To date however, there is still no sufficiently accurate and efficient way for objective monitoring of swallowing behavior in typical daily life environments. Therefore, we have devised a swallowing monitoring system that utilizes a combination of respiratory, acoustical and kinetic signals for more integrated monitoring and assessment of swallowing function. The rationale for the use of respiratory information is twofold: First, it serves as a good marker to detect swallows, since respiratory flow stops during swallowing (deglutition apnea). Secondly, it enables assessment of the coordination between swallowing and breathing, an important airway protection mechanism [28].

This paper mainly focuses on the method by which the new swallowing monitoring system detects swallowing events and assesses swallowing function from collected signal components, i.e., a respiratory flow, swallowing sound, and laryngeal motion. This paper extends the previous research work done by Yagi et al. [47].

In order to evaluate the efficiency and effectiveness of the newly developed monitoring system, we simultaneously monitored the VF measurement in both volunteer subjects and patients with dysphagia. We then compared the results obtained by our system and those by VF. We found that the newly developed method is able to accurately detect swallowing events and yield quantitative indices that may facilitate the assessment of some aspects of swallowing dysfunction.

## Materials and methods

### Recorded components

**Fig. 1:**
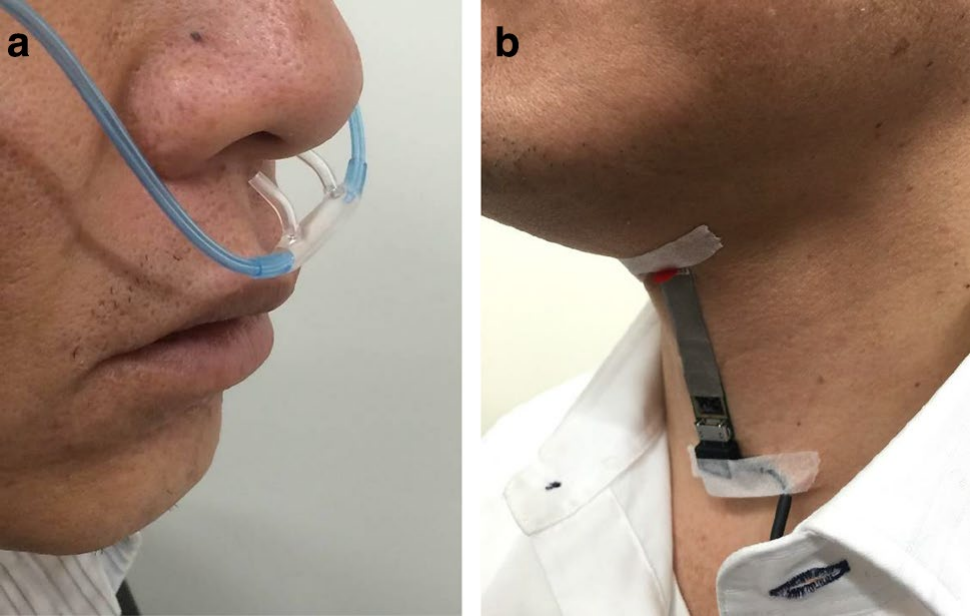
Sensor devices. **a** A nasal cannula-type flow sensor is positioned near the nostril. **b** A piezoelectric sensor is affixed to the surface of the thyroid cartilage using an adhesive tape

Three signal components were recorded by the swallowing monitoring system to detect and evaluate swallowing activity. Respiratory flow was measured by a nasal cannula-type flow sensor (Pro-Tech ProFlow cannula, Sleep Lab Products, USA) and a differential pressure transmitter (KL-17, Nagano Keiki, Japan) and recorded at 1 kHz. Laryngeal motion and swallowing sound were simultaneously recorded using a custom-made, piezoelectric sensor attached on the thyroid cartilage (Fig. 1b). The sensor is a piezoelectric film (the size of the detector is 10 mm × 30 mm), which generates electric charge upon bending. The sensor has a wide dynamic range between 0 Hz and 4 kHz. This sensor was custom made, as to our knowledge there was no commercially available film sensor with a sufficient dynamic range. The signal was amplified and divided into high (>100 Hz)- and low (<100 Hz)-frequency components using high-pass and low-pass preamplifiers, respectively. This cutoff frequency was set to eliminate low-frequency interferences such as heart sounds and muscle artifacts from the (high frequency) sound component [35]. The 100 Hz cutoff frequency also assures the delay characteristics of the low-frequency motion signal component within 30 ms for the frequency range of 0.5–10 Hz, which minimizes the error in estimating the timing of laryngeal elevation in relation to the respiratory phase. Thus, the high-frequency component represents sound signal greater than 100 Hz, whereas the low-frequency component contains both laryngeal motion signal and low-frequency (20–100 Hz) sound signal. However, the power spectrum density of this low-frequency component during swallow typically has a peak at ~1 Hz and decreases toward the audible (>20 Hz) frequency range. Therefore, we assume that the low-frequency component mostly represents the laryngeal motion (as well as infrasound vibration) signal and refer to it as ‘the laryngeal motion signal’ hereafter. The laryngeal motion was recorded at 1 kHz, and the sound signal was recorded at 10 kHz and stored simultaneously with the respiratory signal in a Micro SD card for later analyses. In addition, we recorded the timings of a swallow for later verification using a foot switch to generate TTL-level pulse signals. The signals were analyzed using MATLAB (R2014b, Mathworks, USA) on a 64-bit Windows 8 professional-based computer.

### Experimental protocol

Eleven healthy volunteers (9 males and 2 females, 40.1 ± 10.7 years old) and ten patients with dysphagia (4 males and 6 females, 75.6 ± 9.4 years old) were enrolled in this study. The severity of dysphagia in these patients was assessed using the Food Intake LEVEL Scale (FILS) [12]. Eight patients were at level 3 (swallowing training using a small quantity of food is performed), and two patients were at level 7 (easy-to-swallow food is orally ingested in three meals. No alternative nutrition is given). In seven healthy subjects and in ten patients, swallowing and respiration during videofluoroscopic measurements were simultaneously recorded using two sensor devices (Fig. 1). A nasal cannula-type flow sensor was attached to the nostril (Fig. 1a), and a piezoelectric sensor was placed on the skin surface around the thyroid cartilage with an adhesive tape (Fig. 1b). Each subject was instructed to swallow three types of test food (level 0, level 2, and level 3) and water, two times, during simultaneous swallowing monitoring and VF. A nonionic contrast agent, iopamidol (Oypalomin-370, Konica Minolta, Japan), was mixed into these test foods so that it was diluted twofold (iodine concentration: 185 mg/mL). The physical properties, e.g., viscosity, adhesiveness, and cohesiveness, of the test foods were precisely controlled (Table 1). The composites of level 0, level 2, and level 3 were similar to soft jelly, hard jelly, and paste, respectively. We did not add the contrast agent to the water. This protocol was approved by local ethical committees of Hyogo College of Medicine (No. 1580 and No. 1636), Kyoto University (No. C819 and No. C820), Takahashi Hospital, and Wakakusa Tatsuma Rehabilitation Hospital.

**Table 1:** Test food texture

	Hardness (N/m^2^)	Cohesiveness (J/m^3^)	Adhesiveness
*Coffee taste*			
Level 0	5104 ± 354	14 ± 11	0.262 ± 0.015
Level 2	11,618 ± 846	10 ± 1	0.430 ± 0.060
Level 3	451 ± 16	83 ± 9	0.862 ± 0.015
*Orange taste*			
Level 0	4682 ± 247	40 ± 11	0.246 ± 0.021
Level 2	11,414 ± 596	24 ± 6	0.292 ± 0.017
Level 3	476 ± 19	74 ± 7	0.808 ± 0.012

* Measurement temperature: 20 ± 2 °C

### Respiratory flow component processing

**Fig. 2:**
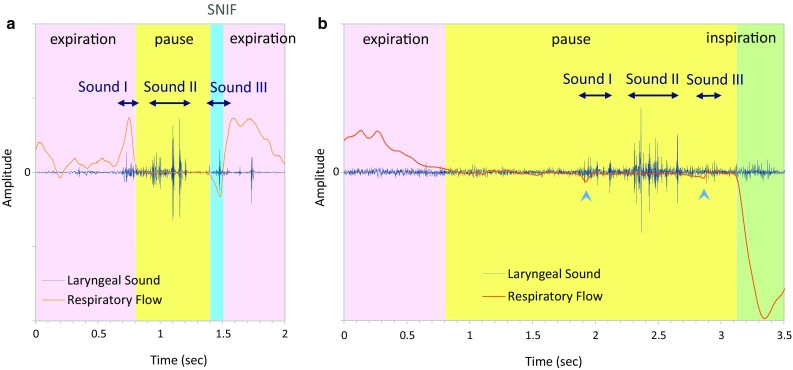
Representative swallow–respiratory coordination patterns are presented together with swallowing sounds (Sounds I, II, and III). *Light pink zone* indicates the expiratory phase, *light yellow zone*, the pause phase, and *light green zone*, the inspiratory phase. **a** Expiration–swallow–expiration pattern. *Blue zone* represents swallow non-inspiratory flow (SNIF). **b** Expiration–swallow–inspiration pattern. Note that small negative pressures (*arrowheads*) are recorded coincident with swallowing sound components

In order to improve the detection accuracy and enhance the functional evaluation of swallowing activity, we analyzed respiratory activity. According to our previous study [47], an analysis using the combination of swallowing sound and breathing information could increase the accuracy of extracting swallows. Furthermore, the analysis of respiratory activity before and after swallowing is particularly useful and important for patients who have limited ventilatory capacity because breathing is closely coordinated with swallowing activity [17, 20, 40]. First, we classified respiratory activity into three phases: inspiration, expiration, and pause. A pause where no respiratory flow signal is detected may be considered as a deglutition apnea, but it might also be a voluntary cessation of breathing; thus, the two must be differentiated based on the presence of characteristic sound and motion (see Sect. 2.5). The deglutition apnea is an important airway defensive reflex to avoid an aspiration, where breathing is temporarily stopped during deglutition. This respiratory cessation period can be considered as a marker to identify swallowing activity. The algorithm for analyzing respiratory phases first searches for inspiratory-to-expiratory (I–E) transition and expiratory-to-inspiratory (E–I) transition. Then, a pause within a breath is detected as a period where the respiratory flow signal falls within a certain range around the zero-flow level. If the pause duration is greater than 0.35 s, it is considered as a candidate of deglutition apnea. Figure 2 shows examples of respiratory phase discrimination before and after swallowing events using developed algorithms. As shown in Fig. 2a, a brief inspiratory flow signal is often recorded immediately after swallowing. However, this is not a true inspiration, but rather it is a negative pressure associated with the relaxation of the pharyngeal constrictor muscle; it is called swallow non-inspiratory flow (SNIF) [3]. To discriminate between a true inspiration and SNIF, we have set a minimal inspiratory time for a negative pressure swing to be considered as an inspiration, and the program searches the next inspiratory activity if the inspiratory time is less than 0.3 s. These parameter values were set empirically.

### Sound component processing

**Fig. 3:**
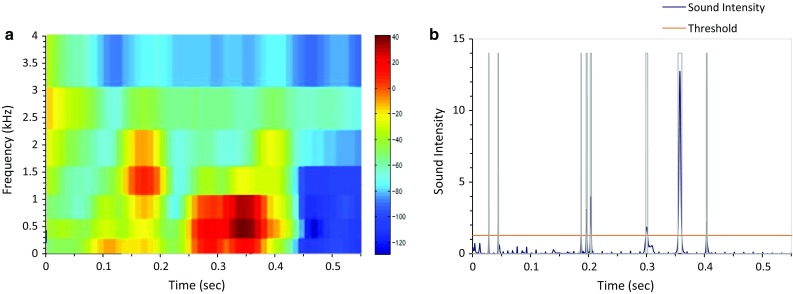
Laryngeal sound is decomposed into **a** the time–frequency domain and **b** sound pulses

Frequency analyses applied to the sound for swallowing detection are shown in Fig. 3. The program first calculates periodogram to estimate the power spectral density. While a simple waveform is a one-dimensional representation of sound, the two-dimensional representation describes the acoustic signal as a time–frequency image. We next applied mel-frequency cepstral coefficients (MFCCs), which is a representation of the short-term power spectrum [6] to those images. This way, we can extract features of swallowing sound characteristics effectively [39]. This technique combines an auditory filter bank with a cosine transform, which provides a rate representation roughly similar to the auditory system. Swallowing sound typically contains a high-frequency component greater than 750 Hz [39], whereas vesicular and bronchial sounds consist of lower (<500 Hz)-frequency sounds [26]. Therefore, the program searches for particular sound data that generated specific high-frequency bands during monitoring. Several frequency decomposition methods have been used for analysis of non-stationary signals such as continuous wavelet transform (CWT) and short-time Fourier transformation (STFT). We used STFT with an epoch duration of 1.5 s and a step size of 0.2 s for presenting the whole data, STFT with an epoch duration of 0.1 s and a step size of 0.01 s for presenting the sound signal within respiratory cessation periods (Fig. 3a), and CWT for feature extraction. The CWT was computed using the Morlet wavelet.

The sound data during respiratory cessation periods are retrieved, and the percent power of 500–2300 Hz frequency bands is calculated for each sound signal during epochs. If the percent power of 500–2300 Hz frequency bands is less than 20 %, then we determined that it is less likely to be a swallow according to the sensor characteristics. The sound signal was then decomposed into pulses to obtain two parameters, the number of pulses and the maximal pulse width (Fig. 3b). We defined and discriminated swallowing sound characteristics from those parameters. If the number of pulses is greater than 20, or if the maximal pulse width is greater than 40 ms, then it is considered to be an artifact or noise.

A normal swallowing sound typically consists of three sound components [46]. The first sound (Sound I) and the third sound (Sound III) are not always detected; however, the second component (Sound II) is consistently and most remarkably audible among three swallowing sound components [25]. Therefore, the program searches the time point of the most prominent sound power peak within each respiratory cessation period to identify the possible Sound II (Fig. 2).

### Detection of swallowing candidate periods

**Fig. 4:**
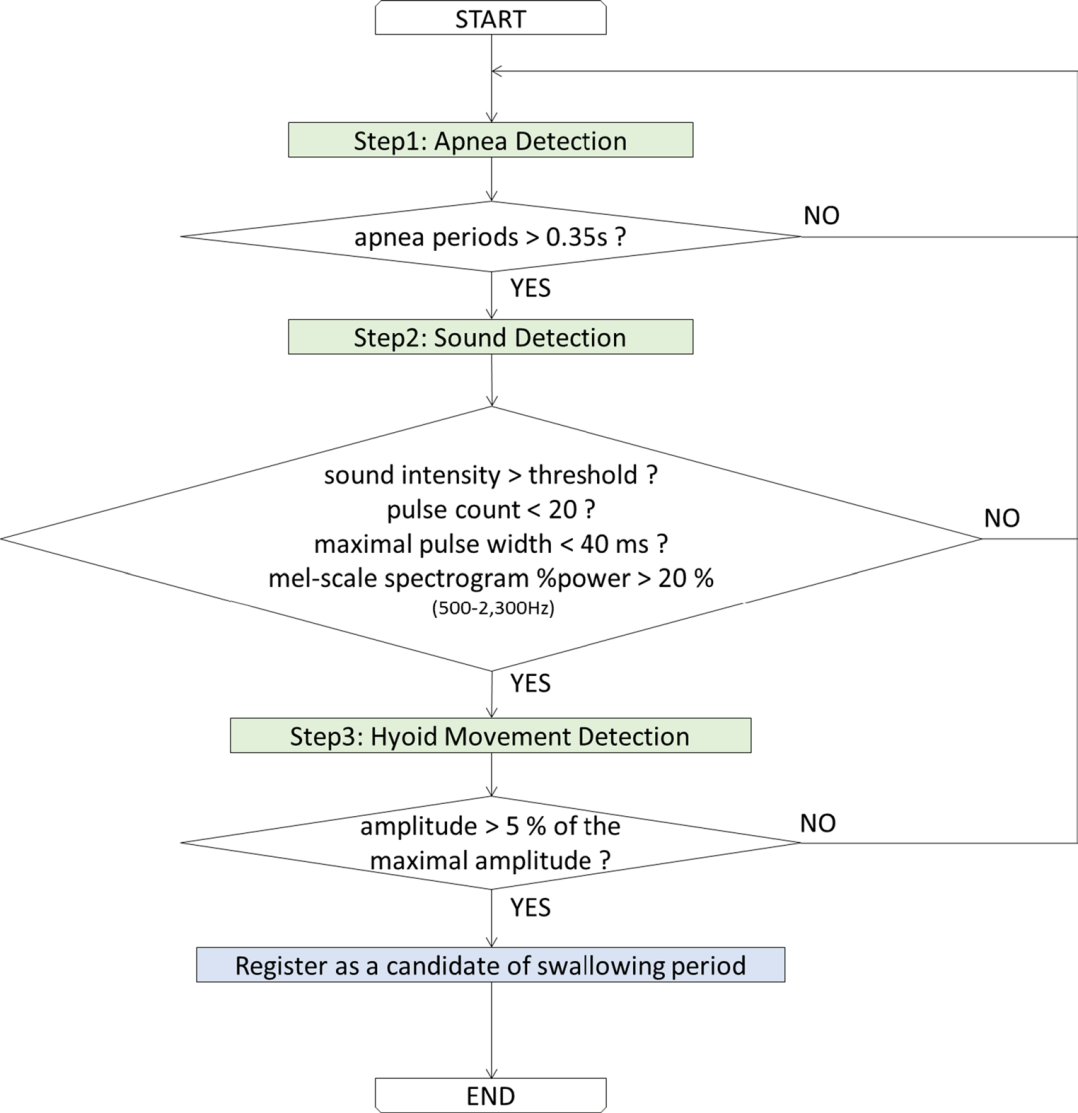
Flow chart of the swallowing detection algorithm

In order to extract swallowing activity, we use deglutition apnea, swallowing sound characteristics, and amplitude of laryngeal motion. We propose a knowledge-based approach to discriminate between swallowing sounds and noises. The swallowing candidate period detection algorithm is shown in Fig. 4.

First respiratory cessation periods (>0.35 s) are extracted (Step 1). If an extracted period contains sound whose intensity is greater than a certain threshold (e.g., noise level + 2 × standard deviation), proceed to further steps (Step 2). In the next step, sound characteristics are analyzed as described above. If the pulse count is <20 counts, the maximal pulse width of which is <40 ms, and mel-scale spectrogram  %power within 500–2300 Hz frequency bands is >20 %, and it is associated with laryngeal motion of amplitude >5 % of the maximal amplitude within the entire record; then, the extracted period is registered as a candidate of swallowing period.

### Laryngeal motion component processing

**Fig. 5:**
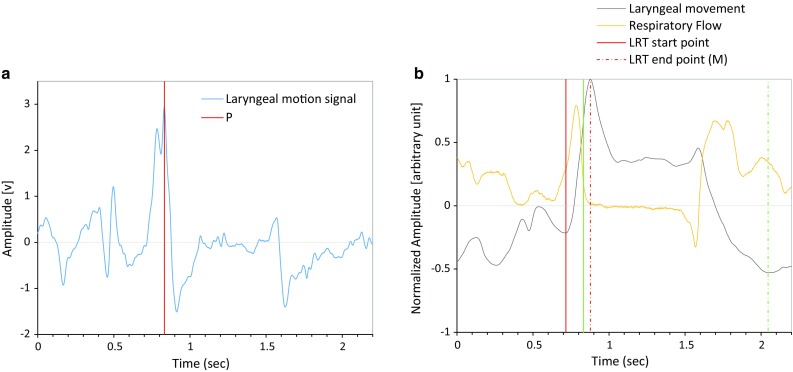
**a** Raw sensor output. The program detects the time point *P* (*red line*) at which the sensor output reaches the peak during the laryngeal elevation and the zero-cross point is searched backward and forward to identify the start point (the trough *T*_1_ in **b**) and end point (the maximum *M* in **b**) of laryngeal rising time (LRT). **b** Integrated sensor signal (*gray*). The *green line P* corresponds to the position of the highest peak in **a**, in which the laryngeal elevation speed becomes maximal. The duration between the trough *T*_1_ (*solid red line*) and the peak *M* (*dashed red line*) is the duration of LRT. The duration between the time point *P* and the trough *T*_2_ is the duration of laryngeal activation duration (LAD)

Evaluating laryngeal motion is critical for assessing the swallowing function. During swallowing, the hyoid bone traces a path upward and forward and then returns to the original position, and the laryngeal prominence (the thyroid cartilage) traces a trajectory closely linked to that of the hyoid bone [41]. Therefore, the motion data are extracted based on the displacement of the laryngeal prominence. Here, we provide two temporal parameters to evaluate the swallowing function. Since the piezoelectric sensor has a differential characteristic against bending, it produces a positive signal associated with the laryngeal elevation and a negative signal associated with the laryngeal descent (Fig. 5a). Therefore, we first integrated the piezoelectric signal to estimate the laryngeal motion (Fig. 5b):

1 $${\text{ILM}} = \int {{\text{LM(}}{\textit{t}}) - \overline{\text{LM}} }$$

where LM is the raw laryngeal motion signal and ILM is the integrated laryngeal motion signal. However, the integrated piezoelectric signal does not completely match the motion of the thyroid cartilage. Therefore, we define two parameters that characterize the shape of the integrated piezoelectric signal and compare them with parameters that characterize the dynamics of swallowing on videofluoroscopy.

### Laryngeal rising time (LRT)

**Fig. 6:**
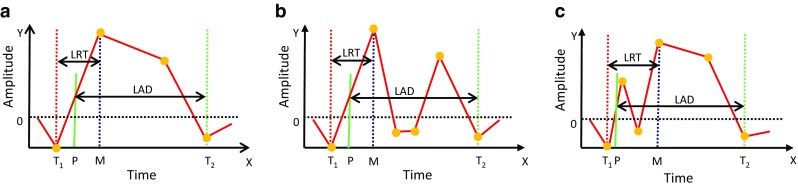
Representative ILM trajectory patterns during swallowing. **a** Standard monotonous laryngeal elevation activity, **b** laryngeal activity drops and passes the zero-cross level after reaching the maximal elevation, and **c** laryngeal activity drops and passes the zero-cross level before reaching the maximal elevation. The duration between the trough *T*_1_ (*red dotted line*) and the peak *M* (*blue dotted line*) is the duration of laryngeal rising time (LRT). The duration between the time point *P* (at which the laryngeal elevation speed becomes maximal) and the trough *T*_2_ is the duration of laryngeal activation duration (LAD)

Slow laryngeal elevation may cause a delay of laryngeal closure for airway protection during the pharyngeal phase of swallowing. Therefore, we define laryngeal rising time (LRT) as the time required for the larynx to elevate to the highest position (Figs. 5, 6).

Within each identified respiratory cessation period, the program first searches for the time point (*P*) at which the sensor output (LM) reaches the highest peak. Due to the differential characteristics of the piezoelectric sensor, this corresponds to the instance when the laryngeal elevation speed becomes maximal. Since the time point *P* corresponds to the onset of pharyngeal swallow, which usually occurs after the onset of respiratory cessation [20], and Sound II is associated with bolus transit [46], we limited the possible position of *P* to be the range between (the onset of deglutition apnea-200 ms) and (Sound_II + 200 ms). Then, we defined *P* as the local maxima within this range (Fig. 5a).

Next, the program searches the zero-cross point backward to estimate the start point of LRT (*T*_1_). If ILM at this zero-cross point has a positive value, then the backward search is continued until the local minima of ILM with a negative value are found. The program then looks for the time point *M* where the larynx reaches the maximal elevation (Fig. 5b). *M* corresponds to the first zero-cross point in LM searched forward from *P*. Finally, LRT is calculated as the duration between *T*_1_ and *M*. Due to the varying structure of swallowing pattern recorded from subjects with different swallowing functions and different postures, several types of ILM patterns can be observed (Fig. 6). Therefore, if LRT is less than 45 ms, then the program finds the second highest LM peak within the range between (the onset of deglutition apnea-200 ms ) and (Sound II + 200 ms) and repeats the LRT calculation until LRT > 45 ms.

### Laryngeal activation duration (LAD)

We next define laryngeal activation duration (LAD) as the duration between the time point *P* and the time point at which the integrated sensor output becomes the trough (*T*_2_) during the descent of the larynx (Figs. 5 and 6). Since LAD represents the duration of the pharyngeal swallow, LAD should be greater than 500 ms; otherwise, the next trough is searched forward.

We set minimal values for LRT and LAD, since ILM sometimes displayed zero-cross activity patterns (Fig. 6b, c). Zero-cross activity patterns were often observed associated with extension and flexion of the head of subjects, since such swallowing maneuvers cause the piezoelectric sensor to bend and generate a signal which overlaps with the laryngeal motion signal.

### Swallowing simulator

**Fig. 7:**
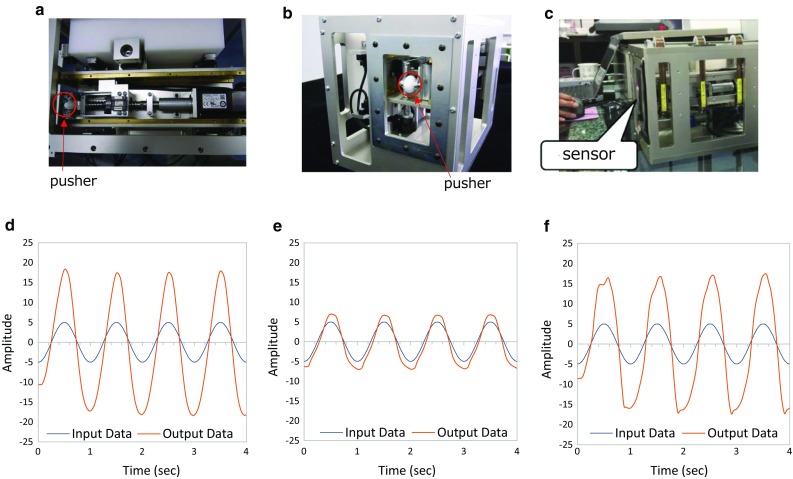
Swallowing simulator that simulates the forward–backward and the anterior–posterior motion of the thyroid cartilage by two linear actuators. **a** Simulator top view, **b** simulator side view, **c** sensor was placed on the pusher, **d** forward–backward input data, **e** anterior–posterior input data, **f** combination input data

In order to assess the characteristics of the newly developed piezoelectric sensor, we created a device that simulates the motion of the thyroid cartilage during swallowing (Fig. 7a–c). The device, hereafter termed ‘swallowing simulator,’ has a motorized pushing mechanism, whose two-dimensional position is controlled by two linear actuators (servomotors and precise screws) that, respectively, simulate forward–backward and upward–downward motion of the thyroid cartilage. Since the major frequency band of laryngeal motion is approximately 1 Hz, we tested responses of the piezoelectric sensor to 1 Hz sinusoidal movements in the forward–backward (Fig. 7d) and upward–downward (Fig. 7e) directions and with a combination of the two (Fig. 7f); we verified that the sensor responded linearly to 1 Hz sinusoidal movements of both the forward–backward and upward–downward directions and also the combination of the two directions, with 40-80ms delay.

### Videofluorographic (VF) measurements

Swallowing function is often assessed by temporal parameters measured using videofluoroscopic images. Among these parameters, we measure the pharyngeal response duration (PRD) [15] and laryngeal elevation delay time (LEDT) [23]. PRD reflects dynamics of the hyoid bone during swallowing. The hyoid bone slowly elevates posteriorly before the initiation of the swallowing reflex and then rapidly starts moving forward upon initiation of the swallowing reflex (pharyngeal swallow) to elevate the larynx. When suprahyoid muscles relax and infrahyoid muscles are activated, the hyoid bone moves backward and downward to return to its original position, and the swallowing reflex is completed. PRD is defined as the duration between the beginning of forward movement and the end of backward and downward movement of the hyoid bone.

LEDT is the time difference between the time when the test food reaches the piriform recess and the time when the larynx reaches the highest position to complete laryngeal closure. LEDT of >0.35 s indicates a risk of aspiration [23]. The prolongation of LEDT may be caused by two independent factors: a delay of the initiation of swallowing reflex and a decrease in laryngeal elevation speed. Since LRT reflects the laryngeal elevation speed, we sought to clarify the relationship between LEDT and PRD.

For spatial measurements, the *Y*-axis was defined as the line connecting the anterior–superior edge of C3 and the anterior–inferior edge of C5, and the *X*-axis was defined as the line perpendicular to the *Y*-axis. Trajectories of the larynx and the hyoid bone were measured by tracking the vocal cord and the anterior ridge of the hyoid bone using a two-dimensional motion analysis software (Move-tr/2D, Library Co. Ltd., Japan).

To compensate for movement of the body, the X–Y coordinates of the anterior–inferior edge of the C4 vertebral body were also measured, which served as the anchor point. Then, the anterior and vertical displacements of the hyoid bone were calculated according to the method described by Kim and McCullough [11].

### Statistics

Occurrence rates of specific coordination patterns between swallowing and breathing in healthy subjects and in patients with dysphagia were compared using Chi-square test. The swallowing characteristics of different food textures/levels were tested using ANOVA. Correlations between the parameters derived from the new device and VF-derived parameters were evaluated by Pearson’s correlation coefficient. LRT and LAD values in healthy subjects and patients were compared using unpaired *t* test, with all data presented as mean ± standard deviation. *P* values were two-sided, and *P* < 0.05 was considered as statistically significant. Statistical analyses were performed using JMP Pro, SAS Institute Inc. (version 12).

## Results

### Accuracy of semiautomatic swallowing detection

The accuracy of semiautomatic swallowing detection was assessed using data from 7 healthy subjects, for whom two speech therapists judged swallow candidates. First, swallowing candidate periods were automatically extracted using the algorithm described in Methods. The automatic detection algorithm picked up 94 swallow candidate periods from 7 subjects, which included 55 test food swallowing periods (confirmed by the timing coincident with foot switch signals) and 39 additional dry (saliva) swallowing candidate periods. Since each subject swallowed test foods eight times, the sensitivity of the automatic swallowing detection algorithm with regard to test food swallows was 55/(7 × 8) = 0.982.

At this point, additional swallowing candidate periods contain false-positive detections (non-swallowing sounds) due to environmental noises, e.g., speech, motion artifacts, and electrical interference. Subsequently, the sound within these respiratory cessation periods (swallowing candidate periods) was played back, and two speech therapists independently judged whether the sound and the laryngeal motion (Fig. 5b) were compatible with a swallow. Each speech therapist judged 28 candidates as dry swallows (true positives) and 11 candidates as non-swallowing sounds (false positives). The judgment perfectly matched, and thus, the Cohen’s kappa coefficient was 1.0. Therefore, the specificity of the automatic swallowing detection algorithm was (94 − 11)/94 = 0.883. When we use only the laryngeal motion characteristics to detect swallows, the sensitivity with regard to test food swallows was 1.0, and the specificity was 0.712.

### Characteristics of swallows

Normal swallows in healthy subjects were accompanied by deglutition apneas, the duration of which was 1441 ± 1152 ms (range 302–5834 ms). It is known that, in general, typical swallows occur during expiration and are followed by expiration (E–SW–E pattern; Fig. 2a). However, in healthy subjects, 4 of 98 swallows occurred during inspiration (I–SW pattern), and 5 of 98 swallows were followed by inspiration (SW–I pattern; exemplified in Fig. 2b). In patients with dysphagia, the duration of deglutition apneas was 2386 ± 2089 ms (range 375–11,599 ms), 7 of 46 swallows occurred during inspiration, and 6 of 46 swallows were followed by inspiration. The occurrence rate of I–SW pattern but not SW–I pattern was significantly increased in the patient group (Chi-squared test of proportion, I–SW: *P* = 0.019 and SW–I: *P* = 0.094).

**Fig. 8:**
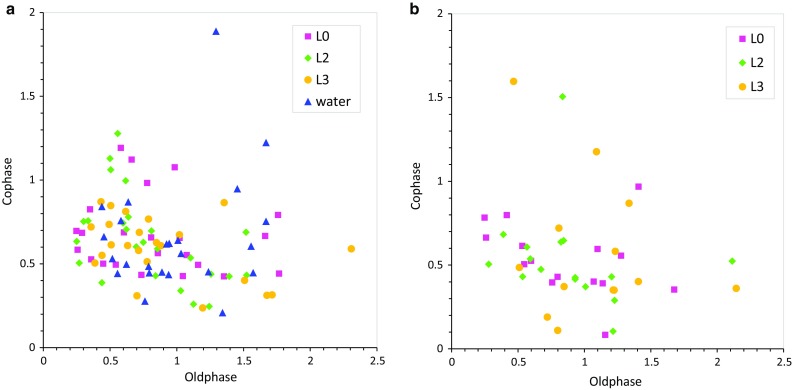
Phase–response curve (co-phase plot) showing how the respiratory rhythm is reset by swallowing events depending on the timing of swallowing. I–SW represents a case where a swallow occurred during inspiration, and SW–I represents a case where a swallow was followed by inspiration. **a** Healthy subjects, **b** patients. One outlier (old phase = 5.85, co-phase = 0.70, L0) is not plotted

The respiratory cycle is reset by a swallowing event, and the timing of initiation of a new inspiration depends on the timing when within the respiratory cycle, the swallowing event occurs [32]. Therefore, we evaluated the phase–response relationship. According to the definition of Paydarfar et al. [32], we calculated the ‘old phase’ as the timing of swallowing from the onset of preceding inspiration and the ‘co-phase’ as the interval between the onset of swallowing and the onset of the subsequent inspiration. Both the old phase and co-phase are normalized by the mean respiratory cycle duration. Figure 8 shows the phase–response curve (co-phase plot), in which the respiratory rhythm was reset by swallowing events. The co-phase was large and variable for swallows initiated near the I–E transition. It should be noted that some swallows occurred after a complete respiratory cycle elapsed (old phase >1); this is thought to be due to the delay in onset of these swallows caused by the chew–swallow complex behavior. These phase–response characteristics did not differ between the healthy subjects and patients.

Normal swallowing sounds in healthy subjects consisted of 6 ± 4 pulses (range 1–20 pulses), the maximal pulse width of which was 8.1 ± 5.8 ms (range 2.4–33.3 ms), and mel-scale spectrogram %power within 500–2300 Hz frequency bands was 71.1 ± 21.9 % (range 21.5–97.3 %). In patients with dysphagia, swallowing sounds consist of 7 ± 4 pulses (range 1–15 pulses), the maximal pulse width of which was 9.1 ± 7.4 ms (range 2.4–34.4 ms), and mel-scale spectrogram %power within 500–2300 Hz frequency bands was 57.3 ± 16.0 % (range 26.4–93.4 %).

### Estimation of swallowing function

Following the semiautomatic swallowing detection, we estimated LRT and LAD and compared those with LEDT and PRD derived from videofluoroscopic image analysis. Since water did not contain a contrast agent, LEDT was not measured for water swallows.

**Fig. 9:**
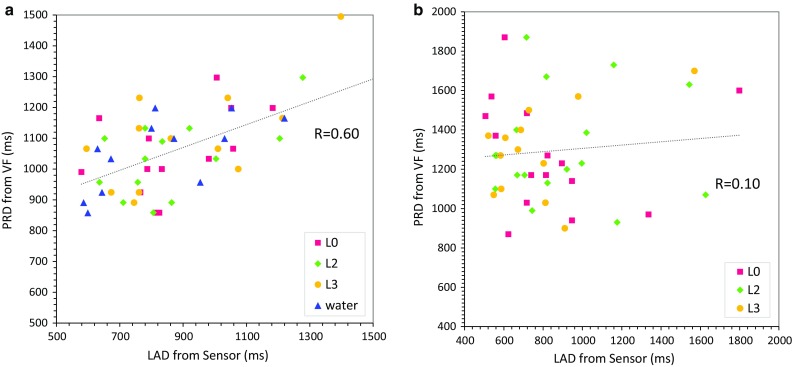
Comparison between laryngeal activation duration (LAD and pharyngeal response duration (PRD). **a** Healthy subjects, **b** patients

In healthy subjects, LRT and LAD were 320 ± 175 ms (range 99–1136 ms) and 959 ± 259 ms (range 579–1699 ms), respectively. On the other hand, LEDT and PRD were 201 ± 56 ms (range 132–297 ms) and 1062 ± 149 ms (range 835–1505 ms), respectively. In patients with dysphagia, LRT and LAD were 465 ± 295 ms (range 151–1583 ms) and 886 ± 311 ms (range 507–1799 ms), respectively. On the other hand, LEDT and PRD were 273 ± 124 ms (range 99–570 ms) and 1292 ± 243 ms (range 870–1870 ms), respectively. The swallowing characteristics of different food textures/levels did not differ in healthy subjects and patients. The correlation coefficient between LRT and LEDT was 0.10 in healthy subjects and 0.07 in patients and that between LAD and PRD was 0.6 in healthy subjects and 0.10 in patients, respectively (Fig. 9). Both LEDT and LRT were significantly prolonged in patients with dysphagia (*P* < 0.0005 and *P* < 0.001, respectively), and the prolongation was more marked in L2 and L3 test foods.

**Fig. 10:**
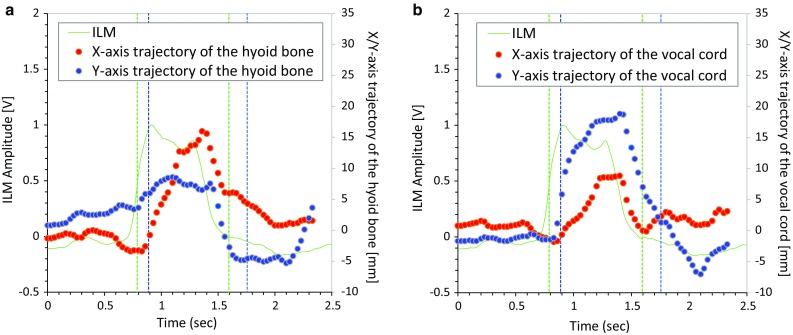
Comparison of integrated laryngeal motion (ILM) signal and trajectories of the hyoid bone (**a**) and the vocal cord (**b**) tracked from videofluoroscopy (VF) images. In each panel, the *blue dots* are the *X*-axis trajectory, the *red dots* represent the *Y*-axis trajectory, and the *green line* is ILM by our monitor device. Start point and end points of laryngeal activation duration (LAD) and pharyngeal response duration (PRD) are shown in *green and blue vertical lines*, respectively

Figure 10 shows the comparison between ILM of the monitor and the trajectories of the hyoid bone (Fig. 10a) and the thyroid cartilage (Fig. 10b) estimated from VF. Since the hyoid bone is connected to the thyroid cartilage via the thyrohyoid muscle, the hyoid bone dynamics are linked to the thyroid cartilage dynamics. However, they do not completely match, because the activity of the thyrohyoid muscle affects the association of their dynamics. Therefore, we sought to clarify the relationship between PRD and LAD. Simultaneous recordings of VF and the piezoelectric sensor revealed that the onset of hyoid elevation is typically delayed against the positive peak of the piezoelectric signal by 100–200 ms.

### Temporal relationships between swallowing sound, motion, and respiratory flow

**Fig. 11:**
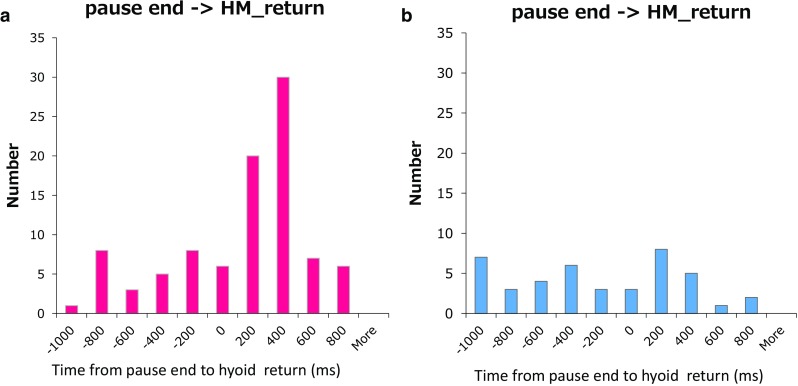
Timing histogram showing when integrated laryngeal motion (ILM) (the laryngeal position) returns to the trough relative to the deglutition apnea. **a** Healthy subjects, **b** patients

We analyzed the temporal relationships between swallowing sound, motion, and respiratory flow according to the spatiotemporal and multi-component analyses using the data from simultaneous VF and monitoring. As illustrated in Fig. 5b, the laryngeal ascent often started before the deglutition apnea. The laryngeal motion speed became maximal at 59 ± 170 ms (range −229 to 539 ms) in healthy subjects and 121 ± 201 ms (range −202 to 579 ms) in patients from the onset of the deglutition apnea, and Sound I occurred between the onset of laryngeal ascension (*T*_1_) and the instance when the laryngeal motion speed became maximal (*P*), indicating that Sound I occurs before the pharyngeal swallow. Sound II occurred at 235 ± 169 ms (range −125 to 799 ms) in healthy subjects and 242 ± 274 ms (range −178 to 1513 ms) in patients from *P*. Assuming that the pharyngeal swallow starts at the time point *P*, this timing suggests that Sound II occurs at the early stage of the pharyngeal swallow. The tail of the bolus was just below the upper esophageal sphincter (UES) at the timing when Sound III was heard. When SNIF was observed, Sound III was typically recorded coincident with SNIF, suggesting that Sound III is associated with the relaxation of the pharyngeal constrictor muscle and the closure of UES. ILM reached the trough *T*_2_ during the descent of the larynx at 25 ± 456 ms (range −1,097 to 796 ms) in healthy subjects and −348 ± 584 ms (range −1359 to 786 ms) in patients relative to the end of the deglutition apnea. In healthy subjects, the trough occurred after the end of the deglutition apnea in 63/98 swallows, indicating that respiration was reinitiated during the descent of the larynx in about 64 % of swallows (Fig. 11a). This phenomenon was less marked (*P* < 0.001) in patients.

## Discussion

In the present study, we developed a new swallowing monitoring system that uses respiratory flow, swallowing sound, and laryngeal motion. We found that LAD was moderately correlated with the VF-derived parameter, PRD; however, this correlation was not observed in patients with dysphagia, suggesting that the motion of the hyoid bone and that of the thyroid cartilage were uncoordinated in these patients. On the other hand, although LRT was not correlated with the comparable VF-derived parameter LEDT, LRT was significantly prolonged in patients with dysphagia. Therefore, LRT may also be a useful parameter for detecting dysphagia. Furthermore, the frequency of the I–SW pattern was significantly increased in patients with dysphagia. These results suggest that the new device may facilitate the assessment of some aspects of swallowing dysfunction, as well as the detection of aspiration risk in specific patient populations.

### Consideration of sound component

The origin of swallowing sound components remains controversial. Vice et al. [46] described the three components of swallowing sound as follows: (1) the initial discrete sound which corresponds to a period when the cricopharyngeus muscle opens, (2) the bolus transit sound which corresponds to the passage of the meal lump to the esophagus, and (3) the final discrete sound which does not always occur. On the other hand, Sato et al. [38] proposed that swallowing sound consists of three sound phases: (1) Sound phase I, may be considered as a closure sound of the epiglottis, (2) Sound phase II, a passage sound through UES, and (3) Sound phase III, an opening sound of the epiglottis. More recently, Moriniere et al. [25] identified three sound components according to the position of the bolus and the anatomic structure in movement: (1) the laryngeal ascension sound when the bolus is located in the oropharynx and/or hypopharynx, (2) the upper esophageal sphincter opening sound where the bolus goes through the sphincter, and (3) the laryngeal release sound when the bolus is located in the esophagus.

**Fig. 12:**
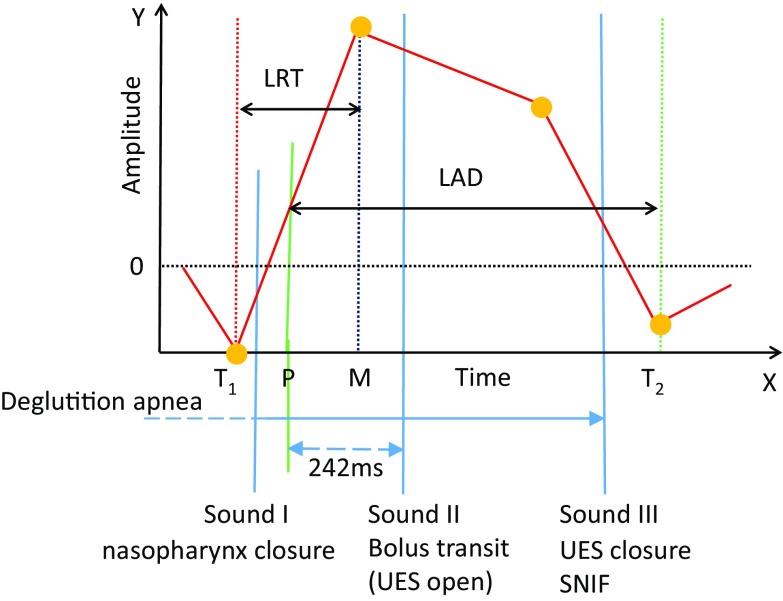
Positions of swallowing sound components and our speculation of their origins

In the present study, we also carefully analyzed the timing of sound occurrence and the position of the bolus. We propose a different interpretation regarding the origin of swallowing sound components, as illustrated in Fig. 12: (1) Sound I occurs during the early (preparatory) phase of the laryngeal ascent before the pharyngeal swallow begins. Therefore, we infer that Sound I corresponds to the closure of the nasopharynx. The bolus is usually located in the oral cavity, but it can be in the oropharynx or the hypopharynx in the case of the chew–swallow complex behavior [21]. (2) Sound II usually occurs 200–250 ms after the onset of the pharyngeal swallow. Since the opening of UES occurs during Sound II, it may contribute to Sound II. However, we think that Sound II is the bolus transit sound mainly through the oropharynx, and the bolus transit sound through UES may not be clearly audible depending on the food consistency, because the tail of the bolus is located just beneath UES at the timing of Sound III, and there is a gap between Sound II and Sound III. In any event, the delay of Sound II from the onset of the pharyngeal swallow indicates dysfunction of the pharyngeal phase of swallowing. (3) We agree with the other theories in that Sound III is the laryngeal release, the opening of the nasopharynx, and UES closure sound, because SNIF is observed coincident with Sound III (Fig. 2b, arrowhead). Interestingly, a small negative pressure is also observed coincident with Sound I (Fig. 2b, arrowhead), suggesting that both Sound I and Sound III are sounds associated with the opening or closure of the upper airway.

### Correspondence between LAD and PRD

Videofluoroscopic studies have shown that coordinated neuromuscular activity of the mouth, pharynx, larynx and esophagus occurs during swallowing. During swallowing, the larynx is elevated by the contraction of suprahyoid muscles and the thyrohyoid muscle, and the epiglottis covers the laryngeal orifice for airway protection. Although these coordinated activities are generated by a reflex and thus stereotypic, the onset of the pharyngeal swallow is variable in its time of occurrence relative to the position of the bolus [18]. This might be one of the reasons why in the present study LRT was poorly correlated with LEDT (Fig. 9a). In contrast, PRD, which does not depend on the position of the bolus, was moderately correlated with LAD (Fig. 9b). PRD is a temporal parameter associated with the motion of the hyoid bone and estimated from VF images, whereas LAD is a temporal parameter associated with the laryngeal motion, and it is estimated from the piezoelectric sensor signal. Although these two parameters are measured by different systems, they are defined to indicate the same temporal property, i.e., the duration between the onset and the offset of the pharyngeal swallow. The duration of the pharyngeal swallow can be one of the parameters defining the swallowing function, since for example the duration of the pharyngeal swallow is prolonged in COPD patients [4].

In the present study, we evaluated whether the onset and the offset of the swallowing reflex, as measured by the two systems, match by simultaneous recordings. The onset of PRD is the time point when the hyoid bone starts the rapid movement anteriorly and upwardly. This time point was coincident with, or slightly (−200 ms) delayed relative to the onset of LAD, which is defined as the time point when the upward laryngeal motion reaches the highest speed (Fig. 10). Since the hyoid bone moves by being tracked by the contraction of suprahyoid muscles, there may be a lag between the hyoid bone movement and the muscle contraction. Therefore, the piezoelectric sensor may detect the muscle contraction associated with the laryngeal elevation and consequently respond slightly earlier than the upward hyoid bone movement. Further, the slow frame rate (30 frames/s) may cause an additional time lag.

We defined the offset of PRD as the time point when the larynx returns to the resting position. The reason why we did not choose the time point when the hyoid bone returns to the onset position was that, since the hyoid bone slightly moves upwardly and posteriorly before the onset of the swallowing reflex, the hyoid bone does not return to the onset position. Further, since the resting position of the larynx is determined by the balance between suprahyoid and infrahyoid muscle tones, and these muscle tones are modified by swallowing activity, it was sometimes difficult to judge whether the larynx had returned to the resting position. Indeed, it has been pointed out that the reliability of parameters tracked on videofluorographic images is poor [45]. Therefore, we added an additional constraint that the thyroid cartilage should be at the locally lowest position when the larynx is at the resting position. As a result, the offset of PRD was coincident with, or slightly (−200 ms) delayed relative to the offset of LAD, which is defined as the time point when the integrated laryngeal motion signal becomes a local minima (Figs. 5, 10). Considering that the inter-rater reliability of temporal VF parameter values, assessed by Cohen’s kappa coefficient, ranged between 0.35 and 0.46 [45], we think that the value of the correlation between PRD and LAD in healthy subjects (*r* = 0.6) was reasonable. However, this correlation was disrupted in patients, suggesting that the linkage between the motion of the hyoid bone and that of the larynx is altered in dysphagic patients.

The speed for food to enter into the pharynx depends on the texture. For instance, L2 and L3 foods enter into the pharynx faster than L0 food, and thus, for dysphagic patients, L2 and L3 foods are more difficult to eat than L0 food. Swallowing characteristics change depending on the food texture, and the alteration is more marked in patients [16]. In the present study, we observed that LRT values of patients were prolonged as compared to those of healthy subjects, and this prolongation was more marked in L2 and L3 foods. Therefore, the use of L2 or L3 foods may be preferable to distinguish swallowing abnormality.

### Coordination between swallowing and breathing

Swallowing and breathing share a common anatomical pathway in the pharynx. Therefore, the airway must be protected against aspiration by a sequence of laryngeal closure, and a precise coordination between breathing and swallowing was controlled by neuronal networks in the medulla. This coordination is critical during swallowing, and its failure can lead to serious consequences. A normal swallowing activity most frequently occurs during the expiratory phase of the breathing cycle, which interrupts the exhalation movement and the breathing resumes with expiration after swallowing has been completed [40]. However, in elderly persons the chance of swallowing occurrence following inspiration and the chance of post-deglutitive resumption of the respiration being inspiration (not expiration) increase [17, 40]. A similar pattern of alteration of the coordination between swallowing and breathing occurs in patients with COPD [10] and Parkinson’s disease [9], which may increase the risk of aspiration.

Martin et al. [20] reported that laryngeal elevation follows the onset of respiratory cessation by 0.19 ± 0.15 s for water swallows. We also observed that the onset of rapid laryngeal elevation associated with swallowing reflex usually follows the onset of respiratory cessation; however, we found that a preparatory slow laryngeal elevation, during which the closure of the oropharynx occurs, is initiated before deglutition apnea (Fig. 5b). Furthermore, we observed that LRT was greater in dysphagic patients, suggesting that this preparatory slow laryngeal elevation is marked in patients. As to the relationship between laryngeal descent and the termination of respiratory cessation, Martin et al. [20] reported that expiration resumes 0.47 ± 0.44 s before the completion of laryngeal descent. We also observed that expiration resumed before the completion of laryngeal descent in a majority of the healthy subjects (Fig. 11a). However, such a phenomenon was less evident in the patients (Fig. 11b). The physiological significance of expiration before the completion of laryngeal descent remains unclear and necessitates further exploration in the future.

The coordination between swallowing and breathing occurs by the interaction of central pattern generators (CPGs) for swallowing and breathing within the brainstem [7, 30]. Bautista et al. [2] proposed that balanced synaptic interaction along the nucleus of the solitary tract (NTS)/Kölliker–Fuse (KF) nucleus axis is pivotal for effective swallowing/breathing coordination, and an imbalance of the synaptic interaction between and within NTS and KF may have an important role in the pathophysiology of swallowing disorders. In the present study, similar to the cases of COPD [10] and Parkinson’s disease [9], the I–SW pattern was more frequently observed in patients with dysphagia. Thus, we suggest that the disordered coordination between swallowing and breathing may be a sensitive and early indicator of a functional abnormality of swallowing CPG and/or an interaction between swallowing and respiratory CPGs. In addition, altered properties of peripheral effector organs, e.g., lung function impairments, would profoundly affect the coordination between swallowing and breathing. Interestingly, CPAP improves respiration–swallowing coordination during sleep [37], and the improvement in respiratory–swallowing coordination results in favorable effects on airway protection and bolus clearance [19]. Therefore, detection of discoordination between breathing and swallowing may lead to early intervention for asymptomatic patients to prevent aspiration.

Swallows can be viewed as external stimuli to the respiratory CPG. The respiratory rhythm is reset by a swallow, and the respiratory phase is shifted. The amount of shift depends on the timing when the swallow occurs within the respiratory cycle. Such phase–response characteristics reflect the internal structure of the respiratory CPG as well as the properties of relay pathways and effector organs (diaphragm, lung, and chest wall) [29, 31]. Paydarfar et al. [32] reported that the interval between the swallowing event and the onset of inspiration is the shortest when swallowing occurs at the E–I transition and largest when swallowing occurs at the I–E transition, in the case of water swallowing. Therefore, swallows at early expiration are the safest with regard to the risk of aspiration. We observed similar phase–response characteristics in both the healthy subjects and patients. The interval between swallowing and subsequent inspirations was highly variable for swallows which occurred near the I–E transition. This may result from the difference in food consistency. In case of level 0 and level 2 test foods (jelly consistency), the interval tended to be prolonged (Fig. 8). Further study is necessary to elucidate factors altering the variation in the phase–response near the I–E transition.

In addition to the autonomic regulation, voluntary and behavioral controls by higher brain centers may affect the coordination between laryngeal motion and breathing activity. For instance, anticipation of the speed of bolus movement may advance or delay the onset of slow laryngeal ascension before the pharyngeal swallow, because depending on the food consistency, subjects can predict the speed of bolus passing through the oropharynx based on their experience. On the other hand, the residue awareness may delay the laryngeal relaxation to prepare for a dry swallowing, or to clear the residual food.

### Technical considerations

In the present study, we adopted a semiautomatic swallowing detection method. The reason why we adopted a semiautomatic rather than a full-automatic detection method was that the sensitivity must be almost 100 % for the clinical assessment of swallowing function. It was reported that the accuracy of the full-automatic swallowing detection method was 82–85 % [8, 39], and we also achieved a similar accuracy; however, it was not sufficient. Although the present study was done while the participants were awake, the device may be used to monitor swallowing while subjects are asleep. Therefore, in a practical situation, several artifacts such as head movement, talking, snoring and electrical interference may further deteriorate the accuracy of swallowing activity detection. In addition, mouth breathing, often observed during the chew–swallow complex behavior, may blunt the respiratory flow signal captured by the nasal cannula-type flow sensor, thereby obscuring the expiratory flow. Therefore, we adopted the semiautomatic detection method to pick up all swallows. We found that the inter-rater variability judged from played back sound and displayed laryngeal motion was extremely small (kappa coefficient = 1.0).

We set the cutoff frequency at 100 Hz to divide sound and motion components. This cutoff frequency might be too high, because the low-frequency component contains both laryngeal motion signal and low-frequency (20–100 Hz) acoustic signal. Lee et al. [14] reported that most of the signal energy measured by accelerometry is contained in the low frequencies, approximately below 100 Hz. However, they suggested that *the accelerometry signal may be primarily attributed to a mechanical rather than acoustic phenomenon*. On the other hand, swallowing sound typically contains a high-frequency component of greater than 750 Hz (see Fig. 1 in Sazonov et al. [39]), and Sarraf-Shirazi et al. [35] use the 100 Hz cutoff frequency to characterize the swallowing sounds recorded in the ear, nose, and on trachea. Therefore, we assumed that this high (>100 Hz)-frequency component is essential in discriminating the swallowing sound from environmental noises. Indeed, speech therapists were able to accurately discriminate swallows by playing back piezoelectric signals above 100 Hz. Therefore, we think that the signal above 100 Hz captures important features of the swallowing sound; however, the cutoff frequency can be optimized by future development.

### Study limitation

Obviously, the sample size in the present study is insufficient to draw a promising conclusion. Further data collection from healthy subjects as well as patients with dysphagia is necessary to improve the detection algorithm and to define normal swallows. In addition, a full-automatic detection method, such as one using pattern recognition methods, needs to be developed in the future.

The coordination between breathing and swallowing is important in the detection of aspiration, although this study was not designed to investigate the effect of aspiration on the parameters. Further study is necessary to elucidate whether the discoordination between breathing and swallowing detects an aspiration event and/or predicts the risk of aspiration.

## Conclusions

In this study, we proposed a novel sound, motion, and air flow recognition technique to detect swallowing events and assess the swallowing function. To our knowledge, this is the first bedside swallowing monitoring system that can assess the swallowing sound, the laryngeal motion during swallowing, and the coordination between swallowing and breathing in a systematic manner. With the device developed in the present study, swallowing activity is semiautomatically detected at a high sensitivity, and the quality of swallows can be assessed from various aspects, i.e., the duration of swallowing reflex, the timing of swallowing sound relative to the laryngeal motion, and the coordination between breathing and swallowing. Therefore, the new device may facilitate the assessment of some aspects of swallowing dysfunction, especially with respect to the coordination between swallowing and breathing.
